# Supplementary material for: Comprehensive Analysis of the NF-YB Gene Family and Expression under Abiotic Stress and Hormone Treatment in Larix kaempferi
Source: Int J Mol Sci. 2023 May 17;24(10):8910. doi: 10.3390/ijms24108910 (PMC10219387; doi:10.3390/ijms24108910)
Supplement: Supplementary file 1 [file ijms-24-08910-s001.zip › Table S1.pdf]

Table S1 .

List of PCR primers used in this study.

Gene Name	Forward primer	Reverse primer
LkNF-YB1	GCTGACCCCCTCAAGATTTA	CCTGTGGCTGCTAGGTCTGT
LkNF-YB3	CCACACTCGGCTTTGAAGATTACG	CCTCCCATGCCGGAAAGCTCCGC
LkNF-YB8	GCCTCTAGTGGCAGTACTGTT	CTACCACTGTCCTCTAGGAT
LkNF-YB13	CCACCATTAATGGCTCCTC	GTCATATTGTAAGCCCCCAT
LkNF-YB14	GCACACTAGGGTTTGAAGAT	TCATGACAGATCATTGCCCTGC
LkNF-YB17	GGGATTGTCCAAGAGCAGGAAC	TCACTCAGATTGTGATTGCGA
